# The Rise of Partisanship and Super-Cooperators in the U.S. House of Representatives

**DOI:** 10.1371/journal.pone.0123507

**Published:** 2015-04-21

**Authors:** Clio Andris, David Lee, Marcus J. Hamilton, Mauro Martino, Christian E. Gunning, John Armistead Selden

**Affiliations:** 1 Department of Geography, The Pennsylvania State University, University Park, Pennsylvania, United States of America; 2 Department of Urban Studies and Planning, Massachusetts Institute of Technology, Cambridge, Massachusetts, United States of America; 3 Sense*able* City Lab, Massachusetts Institute of Technology, Cambridge, Massachusetts, United States of America; 4 Santa Fe Institute, Santa Fe, New Mexico, United States of America; 5 School of Human Evolution and Social Change, Arizona State University, Tempe, Arizona, United States of America; 6 IBM Thomas J. Watson Research Center, Cambridge, Massachusetts, United States of America; 7 Department of Entomology, North Carolina State University, Raleigh, North Carolina, United States of America; 8 United States Senate Budget Committee, Washington, District of Columbia, United States of America; Cinvestav-Merida, MEXICO

## Abstract

It is widely reported that partisanship in the United States Congress is at an historic high. Given that individuals are persuaded to follow party lines while having the opportunity and incentives to collaborate with members of the opposite party, our goal is to measure the extent to which legislators tend to form ideological relationships with members of the opposite party. We quantify the level of cooperation, or lack thereof, between Democrat and Republican Party members in the U.S. House of Representatives from 1949–2012. We define a network of over 5 million pairs of representatives, and compare the mutual agreement rates on legislative decisions between two distinct types of pairs: those from the same party and those formed of members from different parties. We find that despite short-term fluctuations, partisanship or non-cooperation in the U.S. Congress has been increasing exponentially for over 60 years with no sign of abating or reversing. Yet, a group of representatives continue to cooperate across party lines despite growing partisanship.

## Introduction

Americans today are represented by political figures who struggle to cooperate across party lines at an unprecedented rate, resulting in high profile fiscal and policy battles, government shutdowns, and an inability to resolve problems or enact legislation that guides the nation’s domestic and foreign policy [1]. Partisanship has been attributed to a number of causes, including the stratifying wealth distribution of Americans [2]; boundary redistricting [3]; activist activity at primary elections [4]; changes in Congressional procedural rules [5]; political realignment in the American South [6]; the shift from electing moderate members to electing partisan members [7] movement by existing members towards ideological poles [8]; and an increasing political, pervasive media [9].

The individual representative’s role in facilitating partisanship is less clear. Party affiliation significantly shapes a legislator’s voting record [10], [11], so much that in some cases, a change in a legislator’s party affiliation results in an immediate and significant realignment of voting behavior towards the new party agenda [12]. This change is too rapid to be attributable to contemporaneous changes in constituent ideology, indicating a disconnection between the representative and his or her constituency. Party leaders also ensure obedience by offering incentives such as the prospect of assigning a member to a favored committee or promoting legislation crafted by the member to reach final voting stages, i.e. bringing legislation ‘to the floor’ [13]. As many have concluded [1], much is at stake with this type of party-driven arrangement.

Despite party-level pressures, there are incentives for individual representatives to vote with members of the opposite party on issues that are specific to a district’s geography, such as aging populations, natural resource management, veterans’ affairs, or regional concerns [14]. Moreover, regardless of party affiliation, pairwise relationships may form as a result of social interactions such as sponsoring bills, interacting with lobbyists, creating trust networks for communication, sharing ideas, garnering support for initiatives, negotiating provisions and sharing one’s own sense of ethics and orthopraxy. Vote trading, also known as logrolling, is another incentive for cross-party cooperation [15]. Though difficult to quantify because vote trading discussions are not public information, these would result in increased inter-party cooperation on ideological votes.

Given that individuals are persuaded to follow party lines [10–13], while having the opportunity and incentives to collaborate with members of the opposite party [14], [15], our goal is to measure the extent to which legislators tend to form ideological relationships with members of the opposite party. Specifically, we uncover cooperation rates between individual members of Congress, by leveraging a comprehensive dataset of each legislator’s roll call vote decisions in agreement or disagreement with each other legislator during a specific Congress. This process results in a network of Congressional representatives. Such network structures have been shown to predict future re-elections, define intra-Congressional communities and describe temporal dynamics of Congresses [16–21].

In studies that model Congressional representatives as nodes in a network, nodes are connected with an edge based on a given similarity between nodes, such as bill co-authorship or membership on the same committee [16–21]. We connect nodes with similar voting records on individual roll call votes, which represent similarities in ideology. Notably, the network method differs from prevailing legislator partisanship indexing methods [22–24] as the latter require the subjective quantification of each member on a single (liberal-to-conservative) linear scale (i.e. dimension). These dimensions are considered valuable because they temporally correlate with instances of landmark time periods and events in U.S. History [24]. Distinctive and groundbreaking, these dimensions are accepted as standard practice for quantifying polarization, as they serve as a reliable indicator of the political climate.

Yet, above methods are best used to gauge the behavior of entire systems, and not well-suited for discovering interpersonal patterns of agreement forged by pairs of representatives. The network method is able to sidestep the following considerations of the current partisanship measurement tools [22–24]. First, when rating representatives in terms of a chosen vector of decisions deemed important, the index can be (perhaps incorrectly) manipulated to match correlation with events. The actual vote cocktail used to create the index, as well as how this value is transformed to a linear value is not clear to the layperson—perhaps nor to the seasoned social scientist. Also, polarization scale seems to have an arbitrary minimum and maximum that depends on the subjective choices of the creators. Secondly, when the difference between two representatives’ index values is used describe the ideological distance between a pair of representatives, as in [24], false similarities can occur. In this method, the index is centered at zero, signaling neutrality, and increasingly ‘strong’ members of one party (the other party) are increasing positive (negative) numbers. However, two moderate members can each have a zero index, but could actually disagree on every non-procedural issue. Thirdly, indexing methods are described in whole by aggregate measures, such as mean of members’ indexes as indicators of polarization [22–24] which obfuscate the role of the individual. Instead, network methods leverage raw, disaggregate data on each member’s voting behavior to uncover how cross-party pairs form organic relationships in Congress. More drawbacks to traditional index methods, with a focus on of their inability to detect groups, are astutely described in [21].

In this article, we first examine the decline of representatives who agree with representatives of the opposite political party on proposed legislation, and how this lack of collaborative voting reflects changes partisanship over the past 60 years (1949–2012). Our results show how the relative ease of cross-party cooperation in the late 1960s and early 1970s leads to the decoupling of the parties and the rise of a select few individuals who drive high rates of cross-party cooperation. We next discuss the correlation between decreased cooperation and decreasing legislative productivity in the 1990s and 2000s. We finally interpret findings in terms of overall trends in political climate, multiplicative growth processes, public behavior and the implications for the U.S. constituency.

## Materials and Methods

We use roll call vote data from the U.S. House of Representatives from 1949 (commencement of the 81st Congress) to 2012 (adjournment of 112nd Congress) (see Table 1) as provided by the United States Office of the Clerk of the U.S. House of Representatives via Govtrak [26] as described in [27], in a roll call vote, a representative chooses whether to respond (‘yay’/‘nay’) or abstain from voting on a bill or motion. Abstentions are relatively rare, and are counted as ‘nays’, as they do not support the legislation. Most abstentions come from members who are absent or unable to vote on the majority of votes, and have no network connections (i.e. they are not considered). Substantive roll call votes are proposed actions, bills and legislation regarding topics that produce new laws, such as veterans’ benefits, the budget and health insurance. Procedural roll call votes reflect votes on the organization and timing of the agenda [27], such as motioning to recess. We do not discriminate between these types although the latter are often unanimous votes and are largely excluded from the data set.

**Table 1:** Summary Statistics of Congressional Representatives and Voting Records.

Number of Representatives, Starting Year, and Number of Votes for Each Congress					Average Agreements for Different Types of Pairs				Cross-Party (CP) Pair Behavior based on Threshold Value (where Probability Distributions Meet)	
Congress	Starting Year	Democrats	Republicans	Total Votes	Cross-Party Pairs	D-D Pair	R-R Pair	Threshold Value	Cross-Party Pairs Above the Threshold (Cooperators)	Probability of a CP pair Appearing Above the Threshold ^1^
81	1949	269	176	274	90.7	131.0	130.6	124	6383	0.067
82	1951	241	207	180	56.6	80.9	92.3	76	10552	0.106
83	1953	219	221	147	59.4	72.6	91.4	77	6985	0.072
84	1955	236	204	148	64.6	87.9	86.1	80	8427	0.088
85	1957	241	203	193	75.9	101.4	102.5	99	8903	0.091
86	1959	287	159	180	69.9	101.3	103.7	93	6633	0.073
87	1961	273	176	240	93.4	129.0	135.4	125	7548	0.079
88	1963	261	182	231	85.0	123.6	129.4	117	6376	0.067
89	1965	301	142	393	155.9	202.3	216.8	200	7949	0.093
90	1967	251	188	477	211.8	243.8	274.0	257	10029	0.106
91	1969	250	199	443	192.6	214.1	215.1	241	12672	0.127
92	1971	258	187	645	280.5	313.6	336.0	340	11458	0.119
93	1973	248	195	1070	502.1	589.7	590.5	595	12921	0.134
94	1975	294	148	1264	583.5	714.1	732.2	712	9560	0.110
95	1977	293	146	1537	766.4	872.3	934.0	889	10850	0.127
96	1979	280	160	1274	581.1	717.1	769.7	690	11631	0.130
97	1981	246	196	811	395.3	472.2	495.1	482	9830	0.102
98	1983	274	168	905	411.3	578.0	573.2	518	7939	0.086
99	1985	257	182	889	375.0	593.3	566.3	508	5337	0.057
100	1987	263	179	939	409.2	652.3	609.1	563	4807	0.051
101	1989	265	178	904	403.3	609.2	568.2	537	5630	0.060
102	1991	271	170	932	369.3	629.3	593.5	516	3283	0.036
103	1993	261	180	1122	407.1	792.4	794.7	612	1591	0.017
104	1995	207	231	1340	481.2	862.2	1078.1	763	3122	0.033
105	1997	211	232	1187	516.6	813.8	898.3	747	1501	0.015
106	1999	211	225	1214	605.3	903.0	930.6	786	2477	0.026
107	2001	213	226	996	499.4	748.6	782.3	659	1374	0.014
108	2003	208	230	1221	554.0	942.1	992.7	781	455	0.005
109	2005	202	236	1214	533.3	956.0	948.0	766	280	0.003
110	2007	242	205	1876	695.6	1487.3	1376.1	1122	181	0.002
111	2009	261	182	1655	799.4	1336.8	1276.8	1094	1371	0.014
112	2011	200	244	1606	425.3	1137.1	1297.9	838	1508	0.015

^1^ Note: These likelihoods can also be defined as expectations as described in [34].

For all B(n,2) possible pairs of representatives in a given Congress, we count the number of roll call votes where they voted the same way. We tally an agreement when a pair votes either ‘yay’/’yay’ or ‘nay’/’nay’. For example, Congressman A has voted similarly with Congressman B five times more often than with Congressman C in a session, giving the A-B relationship five times the weight of A-C. The result is a B(n,2)–cell, weighted, undirected graph of pair-wise relationships between representatives. Each pair is classified as either “same-party” (SP) if they are members of the same political party, or “cross-party” (CP) if one representative is Republican and the other Democrat. Independents are rare and are included as CP with all other non-Independents. Independents are not listed as super-cooperators due to their tendency to be in a cross-party pair with the majority of Congress. Representative absences are discarded. Agreements are not normalized by total possible votes or any another factor.

## Results

There are a total of 3,424,343 cross-party (CP) pairs (those comprised of a single Republican and a single Democrat) and 2,239,357 same-party (SP) Pairs (those comprised of two Democrats or two Republicans) in the 60 years of our study (Table 1).

**Fig 1:**
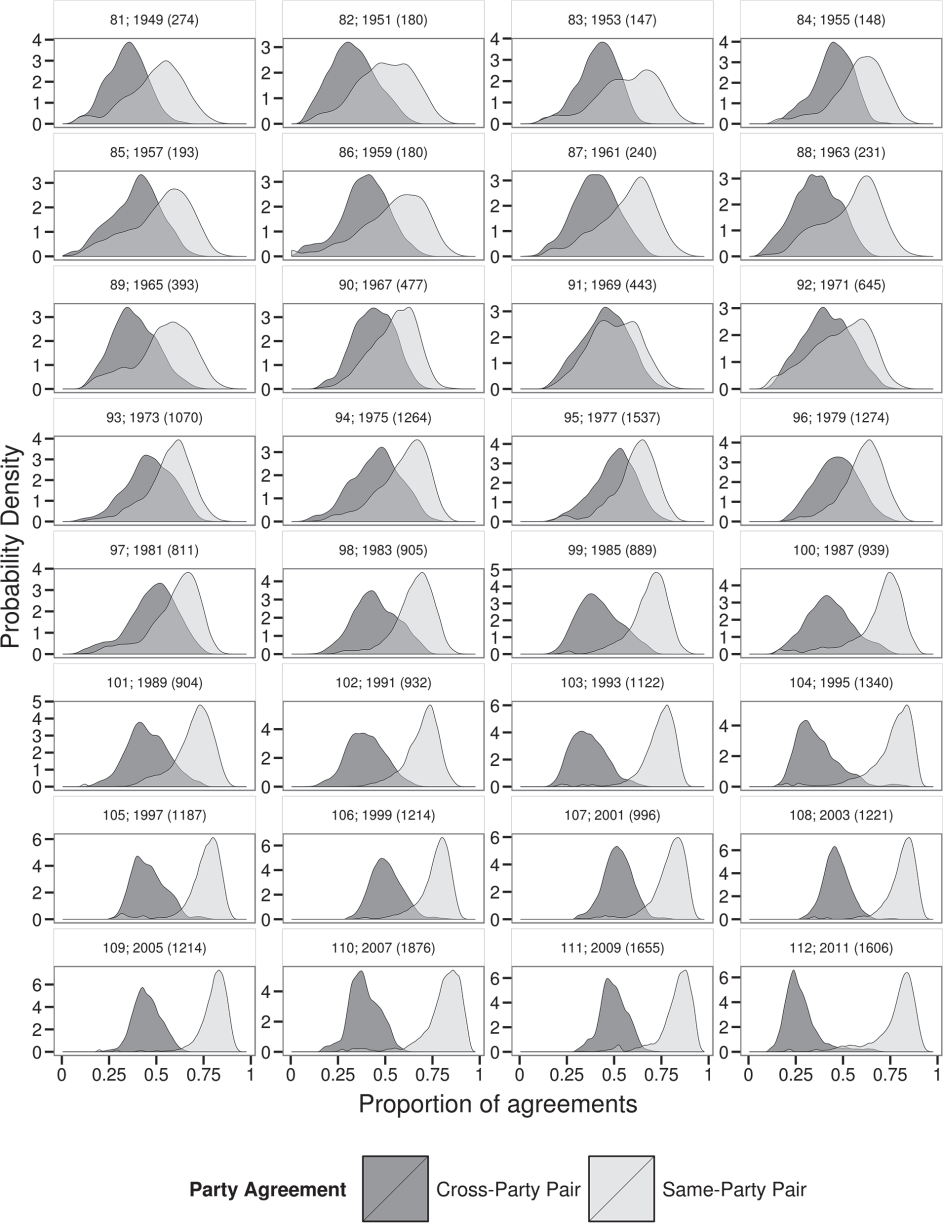
Probability density functions of same-party and cross-party pairs over time. Probability density functions of the number of roll call vote agreements between pairs of the same-party (SP) and those pairs of cross-party (CP) pairs. The plots show the steady divergence of CPs and SP agreement rates over time. Above each distribution is the Congress number (81–112), followed by the year the Congress commenced, and the number of total roll call votes during the two sessions of each Congress. Pairs with few agreements (below the local minima of a consistently- increasing CP distribution), including representatives from Washington D.C., Puerto Rico are removed.

**Fig 2:**
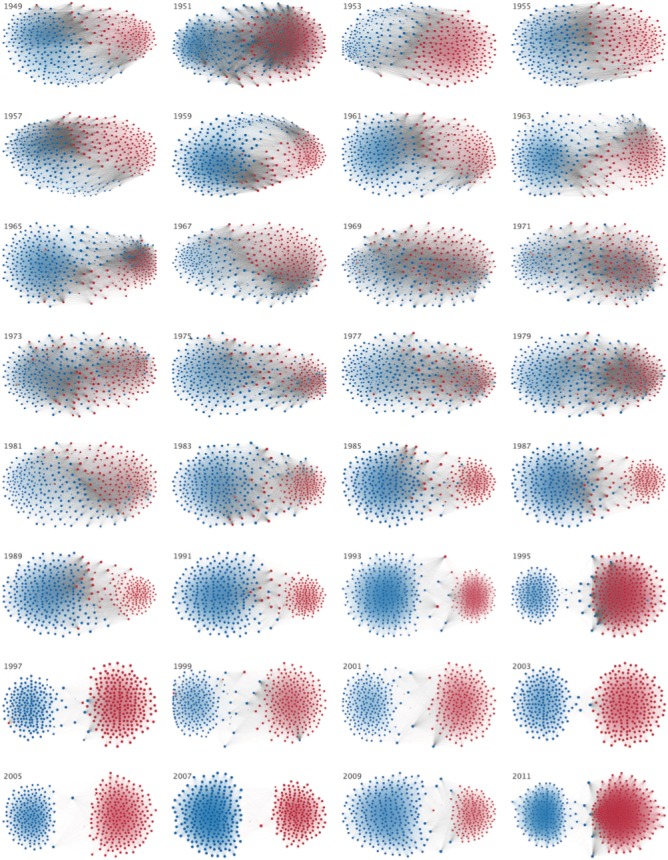
Division of Democrat and Republican Party members over time. Each member of the U.S. House of Representatives from 1949–2012 is drawn as a single node. Republican (R) representatives are in red and Democrat (D) representatives are in blue, party affiliation changes are not reflected. Edges are drawn between members who agree above the Congress’ *threshold value* of votes. The *threshold value* is the number of agreements where any pair exhibiting this number of agreements is equally likely to comprised of two members of the same party (e.g. D-D or R-R), or a cross-party pair (e.g. D-R). Each node is sized relative to its total number of connections; edges are thicker if the pair agrees on more votes. The starting year of each 2-year Congress is written above the network. The network is drawn using a linear-attraction linear-repulsion model with Barnes Hut optimization [33].

For each Congress, a threshold value is defined as the crossing point between dueling frequency distributions (i.e. histograms) of CP and SP pair roll call agreements (Fig 1). For instance, the 109th Congress threshold value is at 766 agreements (Table 1, graphically visible in Fig 1). Although the value itself depends largely on the overall number of roll call votes during a given Congress, the threshold signifies the value at which any random pair who exhibits this number of agreements is equally likely to be a CP or SP pair. A pair found to the right (i.e. with more vote agreements) is more likely to be of the same party (SP); to the left (i.e. with fewer agreements), of two different parties (CP) (Fig 1). CP and SP pairs are nearly indistinguishable from one another in the 91st Congress, but are unmistakably different today (Fig 1). To find the individual legislator’s pairwise agreements over time, we construct a network of representatives (nodes) connected with edges to other nodes if the pair’s vote agreement rate is above the threshold value for that particular Congress (Fig 2). This configuration illustrates the parting of political parties through time while highlighting each individual. (Interactive visualizations are available in S1 Database.)

### Cooperator pairs

**Fig 3:**
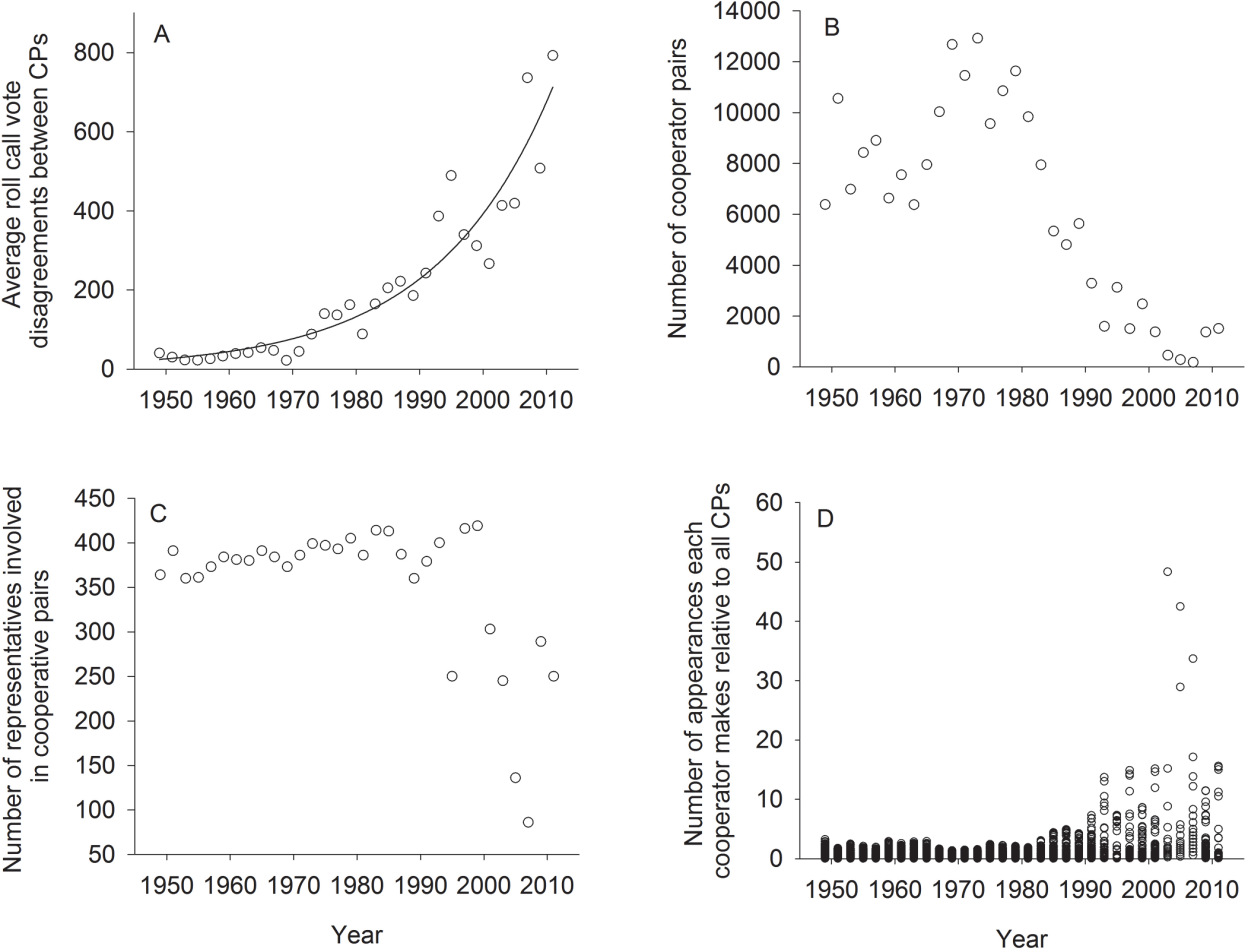
Congressional cooperation rates over time. Four plots of Congressional non-cooperation through time shown as: (a) Average number of roll call vote disagreements between cross-party (CP) pairs as a function of time. (b) The number of cooperator pairs (e.g. cross-party (CP) pairs who agree more often than a random same-party (SP) pair) as a function of time. (c) The number of representatives involved in at least one cooperator pair as a function of time. (d) The number of appearances each cooperator makes relative to all CPs over time evidences super-cooperators from the late 1990s to the present.

Cross-party (CP) pairs above the *threshold value* (Table 1) are distinguished as *cooperators*. These cooperators agree on roll call votes more often than a random SP pair. Cooperator prevalence has decreased by two orders of magnitude from the 1970s to 2000s. From 1967 to 1979, Congress often had over 10,000 cooperators (max: 12,921) and was comprised of at least 10% cooperators (max: 13.4%), i.e. at least 10% of CP pairs agreed on more issues than SP pairs. In comparison, 2001–2010 held fewer than 1,500 cooperators (min: 181) with fewer than 1.5% (min: 0.2%) of CP pairs acting as cooperators (Table 1). Longitudinally, partisanship/non-cooperation has been increasing at an annual rate of about 5% over the last 60 years. The average number of disagreements on roll call votes between CP pairs is increasing exponentially (Fig 3A), as illustrated by an exponential growth model in the form of y = c_0_e^γt^ which exhibits a fit (*F*_*31*_ *= 236*.*22*, *α = 0*.*05*, *R* ^*2*^ *= 0*.*88*, *p < 0*.*0001*). This curve fits the exponential increase of the raw number of votes disagreed upon per session. When vote disagreements are normalized by possible roll call votes, the trend shows high disagreement rates in the 1950s and early 1960s (S1 Fig). Periods of cooperation and non-cooperation align with the findings of [24].

### Super-cooperators

Though cooperator pairs are relatively infrequent today (Fig 3B) the pairs that exist are driven by very few individuals (Fig 3C and 3D). We define a super-cooperator as a legislator who is found in at least 5% of cooperator pairs during a given Congress. Super-cooperators such as Rep. Ralph Hall (D-TX) guide 48% of all cooperator pairs (see S1 Table for each of 86 super-cooperators). Rep. Hall, a senior Democrat from rural North Texas (largest city: Sherman), singlehandedly drove nearly half of all cross-aisle partnerships by agreeing on past the threshold with 220 of the 230 Republicans in the 108th Congress (Table 2). Similarly, Rep. Dan Boren (D-OK), whose Oklahoma district (largest city: Muskogee) shares a border with Rep. Hall, contributed to 42% of all cooperator pairs in the 109th session, by partnering with 119 different Republicans (Table 2). Super-cooperators Rep. Dan Boren (D-OK) and Rep. Robert Cramer (D-AL) together accounted for 71.4% of all cooperator pairs in the 109^th^ Congress. Combined, seven members accounted for 98.3% of all cooperator pairs in the 110^th^ Congress (Fig 3D and S1 Table). Amassing cooperation in the hands of very few legislators is a new phenomenon. Before 1990, the maximum participation for any one legislator in a cooperator pair was less than 5%, and often less than 1%.

**Table 2:** Top Super-Cooperators, Comprising at Least 15% of All Cooperator Pairs in a Specific Congress

Congress	Representative	Total CP Pairs above Threshold (i.e. Cooperators) in Congress	Representative’s Appearances	Appearances as a Percentage of all Cooperator Pairs in the Congress
108	Rep. Ralph Hall [D-TX-4]	455	220	48.351648
109	Rep. Dan Boren [D-OK-2]	280	119	42.50000
110	Rep. Christopher Smith [R-NJ-4]	181	61	33.701657
113	Rep. Jim Matheson [D-UT-4]	521	172	33.013436
109	Rep. Robert Cramer [D-AL-5]	280	81	28.928571
110	Rep. Frank LoBiondo [R-NJ-2]	181	31	17.127072
112	Rep. Jim Matheson [D-UT-2]	1508	235	15.583554
112	Rep. Dan Boren [D-OK-2]	1508	235	15.583554
112	Rep. Mike Ross [D-AR-4]	1508	232	15.384615
108	Rep. Robert Cramer [D-AL-5]	455	69	15.164835
108	Rep. Kenneth Lucas [D-KY-4]	455	69	15.164835
107	Rep. Ralph Hall [D-TX-4]	1374	208	15.138282

Most super-cooperators are Democrats who hail from Texas (12 appearances), Mississippi (7), Alabama (5), Louisiana, Indiana (4 appearances each), Georgia, Kentucky, Oklahoma, Ohio, Pennsylvania and Virginia (3 each). The 104th Congress (1995–1996) had the most super-cooperators (13), all of whom were Democrats, mostly from Southern states. Republican super-cooperator appearances are mostly limited to New York (10), New Jersey (5) and Maryland (4), largely in suburban areas outside New York City and Washington, D.C. This trend may be shifting, as preliminary results from the 113th Congress show that the majority of super-cooperators are Republican representatives from New York and New Jersey.

The few super-cooperators, who hand pick legislation and cooperate with members from each party, despite threat of alienation from his or her party [28], [29], may be today’s hallmark example of carefully representing a constituency. These super-cooperators may earn powerful reputations through single-handedly foraging the dwindling ties across divisive parties.

### Comparison with prevailing statistical methods

We compare the CP pair cooperation rates, produced by the cooperator method, to the DW-NOMINATE multi-dimensional scaling method’s “polarization score”, (the difference in Party means of the first dimension) as well as the “overlap”, (the ideological overlap between the Democratic and Republican Parties) [25] (S2 Fig).

Congresses where CP pairs cooperate, (i.e. appear above the threshold), namely 1949–1983, have a wide cooperator value range and a narrow polarization score domain. These Congresses fall in the 50% all CP pair appearance probabilities, (6.5–13.5% of the full range: 0.02–13.5%) but only in 20% of the “polarization score” range (0.43–0.57 of 0.43–1.09), indicating that these 30+ years would be hard to distinguish when defined by the polarization score index (S2A Fig). The opposite is true for some Congresses between 1993–2011, which post probabilities of appearing above the threshold in a relatively narrow range between 0.02% and 2.0%, of the aforementioned range, while the polarization score ranges liberally between 0.73 and 1.09, thus demarcating these years with more political variability than the cooperator method. In essence, the cooperator method presented here and the DW-NOMINATE polarization score is more sensitive to later years, though the values correlate (r^2^: 0.73). Additionally, the DW-NOMINATE method finds that Congresses commencing in 1951 and 1953 exhibit the least polarization (indexes. 435 and. 433, respectively), while the cooperator method shows that Congresses commencing in 1973 and 1979 were the most cooperative, where each representative had a 13% chance of appearing above the threshold with a member of the opposite party.

A comparison between the DW-NOMINATE’s “overlap” statistic exhibits a better correlation with CP-pair probabilities of appearing above the roll call vote agreement threshold, i.e. being cooperators (r^2^:. 83) (S2B Fig). Still, however, the cooperator method’s 1995–2011 values have a sizable range, while the overlap method produces values with few significant digits: 1995–1999 measured at 0.009, 2011 at 0.007 and 2003–2011 at 0.000, indicating less visible precision. These values are hard to differentiate over time, while the cooperator method assigns more a diverse range of values to Congresses in this range (S2B Fig).

The comparison of the two DW-NOMINATE statistics with the newer cooperator statistics does not indicate that either result is more correct. The cooperator method can add more dimension to the characterization of certain time frames, and the DW-NOMINATE statistics produce more fidelity in other time frames. Yet, we believe that values produced by the cooperator method are *straightforward probabilities* that are simple to explain with the following question: What are the odds that any given representative will be a “cooperator”? This probability is simpler, but more transparent than DW-NOMINATE, which require knowledge of feature space and component analysis to interpret these indexes. Instead, the cooperator method provides a quick overview that can be used across representative governments and other voting-bodies worldwide. The DW-NOMINATE should be a complement to the cooperator method, as it remains beneficial for examining multiple facets of each Congress. For example, it provides multiple descriptive statistics whereas the cooperator method provides few.

### Consensus and public opinion

**Fig 4:**
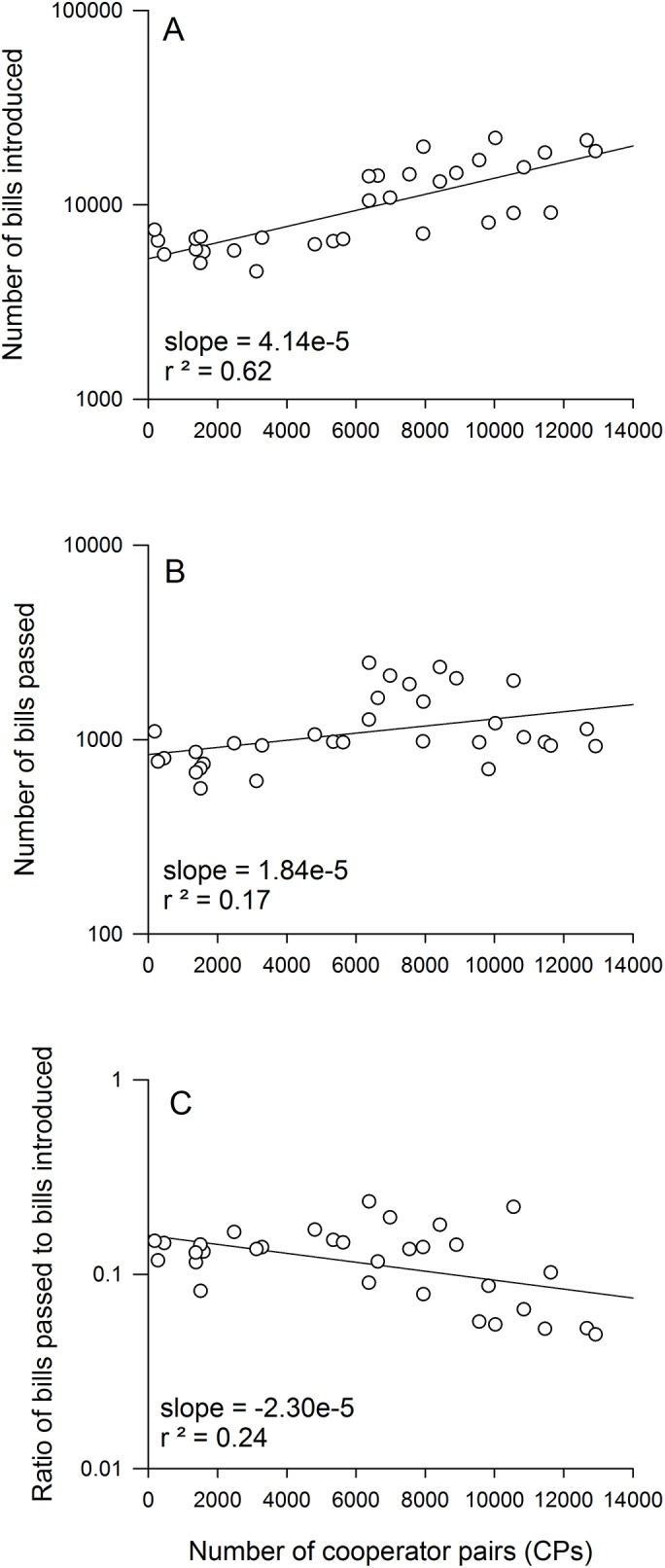
Congressional productivity as a function of cooperation rates. Three plots of Congressional productivity as a function of congressional cooperation show a correlation with: (a) The number of bills introduced during a session. (b) The number of bills passed. (c) The ratio of bills passed to those introduced. Solid lines indicate exponential fits. Data from [30].

Not surprisingly, partisanship correlates with failure to introduce and pass legislation. The number of bills introduced (Fig 4A), bills passed (Fig 4B), and the percentage of introduced bills that pass (Fig 4C) fall exponentially over time, in accordance with a fewer cooperator pairs [30]. The number of bills introduced seems to be most negatively impacted by non-cooperation. This trend is problematic as increase non-cooperation significantly correlates with a decrease in Congressional productivity (Fig 4). Moreover, a decrease in efficiency is also driven by a significant decrease in the number of bills introduced [30], suggesting that increasing non-cooperation stifles Congressional motivation to innovate. This gridlock has resulted in hyperpartisanship and current popular criticism that Congress has recorded its least productive year in 2013 [31].

Moreover, public opinion of Congress has declined simultaneously from 60% favorable rating in the 1960s to a 10% favorable rating today [30] also correlating with more bifurcation in Congress over this time period. We discuss these points further below.

## Discussion

Our analysis shows that Congressional partisanship has been increasing exponentially for over 60 years, and has had negative effects on Congressional productivity. This is particularly apparent in the steady reduction of the number of bills introduced onto the floor, suggesting that the primary negative effect of increasing partisanship is a loss of Congressional innovation.

But why is this pattern of increasing partisanship emerging so strongly? There are complex interactions that drive decision-making and pair-wise relationships in the House of Representatives. Though our data does not support a clear attribution of mechanism other than correlational associations with covariates, we find that polarization is part of a long-term exponential trend implying that non-cooperation *multiplicatively* breeds non-cooperation. In other words, today’s partisan atmosphere may not be a product of recent political splintering (such as the Southern Democrats [32] or the Republican Tea Party Group). Alternatively, such groups may have emerged from a growing shift from cooperation, while simultaneously contributing to the shift. Therefore, while it is incorrect to say that recent divisive political figures are responsible for increasing partisanship, they have actively contributed to it because these are the types of non-cooperative figures and factions that the multiplicative system selects. The exponential increase in non-cooperation shows no indication of slowing, or reversing, and so while Congress has steadily become more non-cooperative over the latter half of the 20th century, this trend seems likely to continue into the future.

This increase in non-cooperation leads to an interesting electoral paradox. While U.S. voters have been selecting increasingly partisan representatives for 40 years, public opinion of the U.S. Congress has been steadily declining. This decline [30] suggests that voters cast their ballots on a local basis for increasingly partisan representatives whom they view as best representing their increasingly partisan concerns, leaving few if any moderate legislators to connect parties for a more cohesive Congress. Elected representatives are increasingly unable to cooperate at a national Congressional level but are re-elected at least 90% of the time, reflecting an evasion of collective responsibility. Voters might believe that highly partisan candidates will ‘tip the scale’ in one party’s favor. However, based on correlations shown here, a partisan candidate may lack cooperation needed to pass legislation. More moderate legislators may have a competitive advantage in negotiating for their party’s legislation.

A fundamental reversal of increasing non-cooperation, over time, might require either a change in local ideological perspectives (resulting in a selective shift to fewer partisan representatives), or a fundamental change in how the electorate votes (from concerns focused on party issues to concerns focused on global effectiveness). Certainly current affairs do not seem to divide potential cooperators, as cross-party relationships peaked in arguably the most tumultuous period in recent U.S. history, marked with numerous political assassinations and Vietnam War and the resignation of President Nixon, as illustrated by others, such as [23–25]. It may be that decreased Congressional social interaction in Washington, D.C., combined with increased telecommunications and commuting to one’s home district, may hamper representatives’ ability cooperate.

The United States is comprised of 435 unique Congressional districts, each with distinct physical geographies, economics, communities, cultures and political ideologies. At one time, these unique constituencies seemed to be represented by a distinct combination of ideologies from the Democratic and Republican Party. Formerly, legislators exhibited a mixture of ideals that resonated across party platforms, allowing each to forge a personal voting fingerprint that reflected the distinctive perspective of his or her unique district and constituency.

Today, districts may remain as socio-economically and geographically unique as in the past, yet representatives have all but lost their personal voting records to complement their individualized constituencies. Instead, Americans today are represented by political figures whose ideological roll call voting record in the U.S. House of Representatives generally resembles one of only *two* types: either a Republican or a Democrat platform, with very little combination. What this unprecedented hyper partisanship will yield for the future of United States foreign and domestic policy is yet to be seen. This work was primarily performed at the Santa Fe Institute.

## Supporting Information

S1 Fig CP vote disagreements divided by all roll call votes, over time. This figure normalizes the number of cross-party pair vote disagreements over the total possible votes in the particular Congress.
(TIF)

S2 Fig Cooperator statistics compared with traditional descriptive statistics. Per Congress, the probability that a legislator is in a CP pair above the threshold (i.e. a cooperator) correlates with two DW-NOMINATE statistics: *political partisanship* and *overlap*, with different dynamics over time. Data from [24].
(TIF)

S1 Database (DOCX)

S1 Table Super-cooperators in cooperator pairs, ordered by percentage of appearances. (DOCX)
