# Supplementary figures and images for: The Rise of Partisanship and Super-Cooperators in the U.S. House of Representatives
Source: PLoS One. 2015 Apr 21;10(4):e0123507. doi: 10.1371/journal.pone.0123507 (PMC4405569; doi:10.1371/journal.pone.0123507)

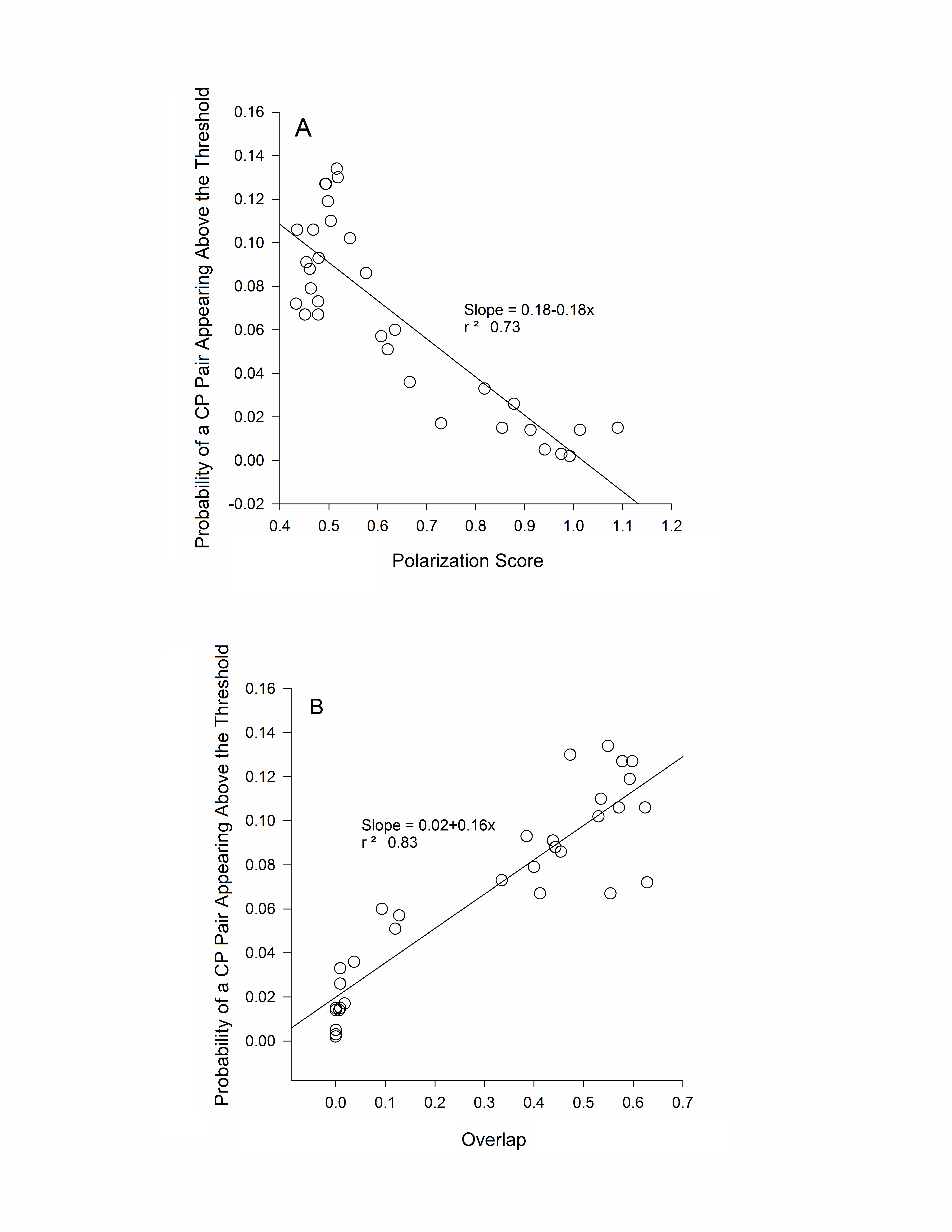

Supplement: S2 Fig — Per Congress, the probability that a legislator is in a CP pair above the threshold (i.e. a cooperator) correlates with two DW-NOMINATE statistics: political partisanship and overlap, with different dynamics over time. Data from [24]. (TIF) [file pone.0123507.s002.tif]
