# Supplementary material for: The Rise of Partisanship and Super-Cooperators in the U.S. House of Representatives
Source: PLoS One. 2015 Apr 21;10(4):e0123507. doi: 10.1371/journal.pone.0123507 (PMC4405569; doi:10.1371/journal.pone.0123507)
Supplement: S1 Database — (DOCX) [file pone.0123507.s003.docx]

**External Database S1**: This database is a website where users can explore each network in detail at <http://www.mamartino.com/projects/rise_of_partisanship>. For each Congress, network nodes are labeled by representative last name and state of his or her district.
