# Supplementary material for: The Rise of Partisanship and Super-Cooperators in the U.S. House of Representatives
Source: PLoS One. 2015 Apr 21;10(4):e0123507. doi: 10.1371/journal.pone.0123507 (PMC4405569; doi:10.1371/journal.pone.0123507)
Supplement: S1 Table — (DOCX) [file pone.0123507.s004.docx]

**Table S1.**

| Congress | Representative | Total CP Pairs above Threshold (Cooperators) in the Congress | Representative’s Appearances | Appearances as a Percentage of all Cooperator Pairs in the Congress |
| --- | --- | --- | --- | --- |
| 108 | Rep. Ralph Hall [D-TX-4] | 455 | 220 | 48.351648 |
| 109 | Rep. Dan Boren [D-OK-2] | 280 | 119 | 42.5 |
| 110 | Rep. Christopher Smith [R-NJ-4] | 181 | 61 | 33.701657 |
| 113 | Rep. Jim Matheson [D-UT-4] | 521 | 172 | 33.013436 |
| 109 | Rep. Robert Cramer [D-AL-5] | 280 | 81 | 28.928571 |
| 110 | Rep. Frank LoBiondo [R-NJ-2] | 181 | 31 | 17.127072 |
| 112 | Rep. Jim Matheson [D-UT-2] | 1508 | 235 | 15.583554 |
| 112 | Rep. Dan Boren [D-OK-2] | 1508 | 235 | 15.583554 |
| 112 | Rep. Mike Ross [D-AR-4] | 1508 | 232 | 15.384615 |
| 108 | Rep. Robert Cramer [D-AL-5] | 455 | 69 | 15.164835 |
| 108 | Rep. Kenneth Lucas [D-KY-4] | 455 | 69 | 15.164835 |
| 107 | Rep. Ralph Hall [D-TX-4] | 1374 | 208 | 15.138282 |
| 112 | Rep. Collin Peterson [D-MN-7] | 1508 | 226 | 14.986737 |
| 105 | Rep. James Traficant [D-OH-17] | 1501 | 223 | 14.856762 |
| 107 | Rep. Kenneth Lucas [D-KY-4] | 1374 | 201 | 14.628821 |
| 105 | Rep. Ralph Hall [D-TX-4] | 1501 | 214 | 14.257162 |
| 105 | Rep. Virgil Goode [D-VA-5] | 1501 | 210 | 13.990673 |
| 110 | Rep. John Barrow [D-GA-12] | 181 | 25 | 13.812155 |
| 103 | Rep. Benjamin Gilman [R-NY-20] | 1591 | 218 | 13.702074 |
| 103 | Rep. Constance Morella [R-MD-8] | 1591 | 207 | 13.010685 |
| 110 | Rep. Joe Donnelly [D-IN-2] | 181 | 22 | 12.154696 |
| 107 | Rep. Robert Cramer [D-AL-5] | 1374 | 164 | 11.935953 |
| 111 | Rep. Walter Minnick [D-ID-1] | 1371 | 157 | 11.451495 |
| 111 | Rep. Bobby Bright [D-AL-2] | 1371 | 156 | 11.378556 |
| 105 | Rep. George Miller [R-CA-7] | 1501 | 170 | 11.325783 |
| 112 | Rep. Jason Altmire [D-PA-4] | 1508 | 169 | 11.206897 |
| 113 | Rep. Michael G. Grimm [R-NY-11] | 521 | 56 | 10.74856 |
| 103 | Rep. Gene Taylor [D-MS-5] | 1591 | 167 | 10.496543 |
| 112 | Rep. John Barrow [D-GA-12] | 1508 | 158 | 10.477454 |
| 113 | Rep. Jon Runyan [R-NJ-3] | 521 | 50 | 9.596929 |
| 111 | Rep. Parker Griffith [R-AL-5] | 1371 | 131 | 9.555069 |
| 103 | Rep. Ralph Hall [D-TX-4] | 1591 | 149 | 9.365179 |
| 113 | Rep. Peter T. King [R-NY-2] | 521 | 47 | 9.021113 |
| 103 | Rep. Charles Stenholm [D-TX-17] | 1591 | 141 | 8.862351 |
| 108 | Rep. Rodney Alexander [D-LA-5] | 455 | 40 | 8.791209 |
| 111 | Rep. Harry Mitchell [D-AZ-5] | 1371 | 120 | 8.752735 |
| 106 | Rep. Virgil Goode [D-VA-5] | 2477 | 214 | 8.639483 |
| 106 | Rep. James Traficant [D-OH-17] | 2477 | 212 | 8.55874 |
| 110 | Rep. Baron Hill [D-IN-9] | 181 | 15 | 8.287293 |
| 106 | Rep. Ralph Hall [D-TX-4] | 2477 | 204 | 8.235769 |
| 103 | Rep. William Tauzin [D-LA-3] | 1591 | 128 | 8.045255 |
| 113 | Rep. Frank LoBiondo [R-NJ-2] | 521 | 41 | 7.869482 |
| 105 | Rep. Gene Taylor [D-MS-5] | 1501 | 113 | 7.528314 |
| 106 | Rep. Kenneth Lucas [D-KY-4] | 2477 | 183 | 7.387969 |
| 111 | Rep. Travis Childers [D-MS-1] | 1371 | 101 | 7.366885 |
| 106 | Rep. George Miller [R-CA-7] | 2477 | 182 | 7.347598 |
| 104 | Rep. Ralph Hall [D-TX-4] | 3122 | 228 | 7.303011 |
| 104 | Rep. William Tauzin [D-LA-3] | 3122 | 228 | 7.303011 |
| 104 | Rep. Michael Parker [D-MS-4] | 3122 | 228 | 7.303011 |
| 104 | Rep. Nathan Deal [D-GA-9] | 3122 | 228 | 7.303011 |
| 104 | Rep. Gregory Laughlin [D-TX-14] | 3122 | 228 | 7.303011 |
| 102 | Rep. Benjamin Gilman [R-NY-22] | 3283 | 239 | 7.279927 |
| 111 | Rep. Gene Taylor [D-MS-4] | 1371 | 99 | 7.221007 |
| 104 | Rep. Preston Geren [D-TX-12] | 3122 | 225 | 7.206919 |
| 110 | Rep. Jason Altmire [D-PA-4] | 181 | 13 | 7.18232 |
| 104 | Rep. Gillespie Montgomery [D-MS-3] | 3122 | 222 | 7.110826 |
| 105 | Rep. Charles Stenholm [D-TX-17] | 1501 | 106 | 7.061959 |
| 104 | Rep. William Brewster [D-OK-3] | 3122 | 220 | 7.046765 |
| 102 | Rep. Frank Horton [R-NY-29] | 3283 | 223 | 6.792568 |
| 102 | Rep. Sherwood Boehlert [R-NY-25] | 3283 | 221 | 6.731648 |
| 102 | Rep. Sedgwick Green [R-NY-15] | 3283 | 220 | 6.701188 |
| 107 | Rep. Charles Stenholm [D-TX-17] | 1374 | 90 | 6.550218 |
| 111 | Rep. Glenn Nye [D-VA-2] | 1371 | 89 | 6.491612 |
| 107 | Rep. Ike Skelton [D-MO-4] | 1374 | 89 | 6.477438 |
| 104 | Rep. Charles Stenholm [D-TX-17] | 3122 | 201 | 6.438181 |
| 113 | Rep. Rodney P. Frelinghuysen [R-NJ-11] | 521 | 33 | 6.333973 |
| 104 | Rep. Gary Condit [D-CA-18] | 3122 | 196 | 6.278027 |
| 107 | Rep. Constance Morella [R-MD-8] | 1374 | 85 | 6.186317 |
| 104 | Rep. Gene Taylor [D-MS-5] | 3122 | 192 | 6.149904 |
| 111 | Rep. Frank Kratovil [D-MD-1] | 1371 | 84 | 6.126915 |
| 110 | Rep. Brad Ellsworth [D-IN-8] | 181 | 11 | 6.077348 |
| 102 | Rep. Constance Morella [R-MD-8] | 3283 | 199 | 6.061529 |
| 107 | Rep. Tim Holden [D-PA-6] | 1374 | 80 | 5.822416 |
| 113 | Rep. Tom Cole [R-OK-4] | 521 | 30 | 5.758157 |
| 109 | Rep. Collin Peterson [D-MN-7] | 280 | 16 | 5.714286 |
| 104 | Rep. James Traficant [D-OH-17] | 3122 | 169 | 5.413197 |
| 104 | Rep. James Hayes [D-LA-7] | 3122 | 169 | 5.413197 |
| 106 | Rep. Constance Morella [R-MD-8] | 2477 | 134 | 5.40977 |
| 113 | Rep. Collin Peterson [D-MN-7] | 521 | 28 | 5.37428 |
| 105 | Rep. Robert Cramer [D-AL-5] | 1501 | 80 | 5.32978 |
| 108 | Rep. James Leach [R-IA-2] | 455 | 24 | 5.274725 |
| 106 | Rep. Michael Forbes [R-NY-1] | 2477 | 130 | 5.248284 |
| 103 | Rep. Sherwood Boehlert [R-NY-23] | 1591 | 83 | 5.216845 |
| 102 | Rep. Hamilton Fish [R-NY-21] | 3283 | 169 | 5.147731 |
| 103 | Rep. Earl Hutto [D-FL-1] | 1591 | 81 | 5.091138 |
| 109 | Rep. Lincoln Davis [D-TN-4] | 280 | 14 | 5.000000 |
